# Coping with COVID-19 pandemic: reflections of older couples living alone in urban Odisha, India

**DOI:** 10.1017/S1463423621000207

**Published:** 2021-11-03

**Authors:** Pranab Mahapatra, Krushna Chandra Sahoo, Shyama Desaraju, Sanghamitra Pati

**Affiliations:** 1 Department of Psychiatry, Kalinga Institute of Medical Sciences, Bhubaneswar, Odisha, India; 2 Regional Medical Research Centre, Indian Council of Medical Research, Bhubaneswar, Odisha, India

**Keywords:** coping, COVID-19, healthy aging, India, LMIC, longevity, older adults, pandemic, SARS-CoV-2, qualitative

## Abstract

**Aim:**

We explored the ‘coping reflections’ of elderly couples living alone (without any other family members) during the COVID-19 pandemic in urban Odisha, India.

**Background:**

Evidence worldwide suggests that older people are at increased risk from COVID-19 adverse outcomes and experience greater stress. In our previous community-based study urban dwelling, particularly elderly participants, and living alone reported higher pandemic-associated health care challenges than their rural and residing-with-family counterparts. We intended to explore how the elderly couples living alone coped through this challenging yet stressful situation during the COVID-19 pandemic and what were their key strategies adopted toward this.

**Methods:**

We conducted telephonic in-depth interviews (IDIs) with 11 urban elderly couples living alone in Bhubaneswar city of Odisha, India using a semi-structured interview guide. All IDIs were digitally recorded, transcribed into the original language, and translated to English. We used a thematic approach for analysis.

**Findings:**

Four themes emerged: (1) Risk appraisal and feeling vulnerable; (2) Safeguarding against COVID-19; (3) Managing routine health care and emergency; and (4) Pursuing mental and psychological well-being. Although fear, anxiety, and loneliness were continuing stressors, many of them learnt to adapt and emerge resilient with the evolving situation. Various elements at the individual, family, community, and organizational levels were conducive to better coping. The companionship and complementary support of spouse, self-health literacy, and digital efficacy, virtual connectedness with family and friends, availability of community pharmacy and diagnostic services in the vicinity, support of neighbors, reengaging with creative leisure time activity, and assurance of a responsive administration at the time of emergency helped them to cruise through the pandemic. Furthermore, watching the re-telecast of prime time serials made these elderly fondly remember their own youth time memories. Self-health monitoring, indoor physical exercise, spiritual practices, continuation of previous prescription, telephonic advice of physicians were add-on strategies that facilitated their physical and psychological well-being during the pandemic.

## Background

Evidence worldwide has shown that older people are at increased risk from COVID-19 due to compromised immunity, which reduces their resistance to infection and increases recovery time (Rahman *et al.*, 2020; Dubey *et al.*, 2020). Elderly fatalities were found to be higher than younger people (Cerasoli, 2020). In addition to ageism and fragility, the presence of preexisting comorbidities increases the risk of infection with COVID-19, disease severity, and mortality (Kessler & Bowen, 2020). Many countries around the globe have made specific recommendations for the elderly to remain in quarantine for extended periods, despite the psychological stress of isolation (Javadi & Nateghi, 2020). Prior research shows that isolation strategies have a disproportionate impact on the elderly and that their resilience varies significantly in a community with differential responses (Fraser *et al.*, 2020; Javadi & Nateghi, 2020; Killgore *et al.*, 2020; Armitage & Nellums, 2020). The elderly who had a well-established social support system and engaged in outside activities showed greater resilience than those who survived without much support (Killgore *et al.*, 2020; Armitage & Nellums, 2020).

The number of elderly couples living without any other family members (living alone) in India is increasing over time, fending for themselves and relying mainly on assistance from home helpers or family members and local physicians (Vahia & Shah, 2020). The national lockdown started on 24 March 2020 in response to the COVID-19 pandemic and lasted until 31 May 2020 in India (Rattan *et al.*, 2020). Several administrative policies, such as the provision of financial support and social security services, have been placed to resolve the healthcare needs of elderly people in India during the pandemic (Kamate *et al.*, 2020; Vahia & Shah, 2020).

COVID-19-related mandatory stay-at-home isolation could have affected the lifestyle of the elderly to quite some extent. Our previous community-based studies on COVID-19 showed that urban participants, particularly elderly people, reported higher pandemic-associated care challenges compared to rural respondents (Pati *et al.*, 2020; Sahoo *et al.*, 2020). In addition, elderly couples living alone have expressed greater difficulties in terms of access to health care and logistic support (Pati *et al.*, 2020; Sahoo *et al.*, 2020). The lived experiences, challenges encountered, and coping strategies during such stressful situations could provide valuable insights toward designing an aging-focused health preparedness plan for any pandemic or similar emergencies.

With this view, in the present study, we explored how the elderly couples living alone in urban India managed through the COVID-19 pandemic and intended to garner an in-depth understanding of their ‘coping reflections’.

## Methods

### Study design, settings, and participants

A reflective narrative approach was adopted to describe the elderly couples’ coping experiences during the COVID-19 pandemic. The study was conducted in Bhubaneswar, the capital city of Odisha, an eastern state in India. The initial COVID-19 case was predominantly being reported from Bhubaneswar, one of the most affected urban municipalities in the state (Government of Odisha, 2020).

Our study was nested within a larger community-based study on noncommunicable disease NCD management during COVID-19 pandemic in Odisha (Pati *et al.*, 2020; Sahoo *et al.*, 2020). In our community-based research, a total of 88 participants from urban areas were 65 years of age and above. Among them, 15 elderly couples living without any other family members were found, with either spouse being 65 years of age or older and having at least 1 chronic condition.

We approached 15 elderly couples and appraised them of the study objective and manner of data collection. Two couples expressed inability owing to lack of interest in the study. Out of 13 couples (*n* = 26) who agreed to participate; we were able to complete interviews with 11. The rest two interviews remained incomplete as the couples had some other engagements coinciding with the interview and could not give time.

### Data collection

Due to the limitations of COVID-19, we preferred telephonic interviews with the elderly couples during the last week of June to the middle of July 2020. Eleven in-depth interviews (IDIs) were undertaken telephonically using a semi-structured, open-ended interview guide (Table 1). The interviews were scheduled as per the day and time convenient for the participants. The first author (PM), a practicing psychiatrist having experience in aging research and a native of the study settings conducted all the interviews. The conversations were carried out in the local language familiar to the participants, in this case, ‘Odia’. Only PM (who was under self-isolation) and the interviewee couple were involved in the virtual encounters to ensure privacy and confidentiality.

**Table 1.** In-depth interview guide

1. What was your daily health-related activities? Probes: diet, exercise, meditation, or any other
2. How were your health issues managed before the pandemic? Probes: routine and emergency consultations, daycare, chemotherapy, or physiotherapy
3. What were your apprehensions toward the COVID-19 pandemic?
4. How did the pandemic affect health care? Probes: consultations, emergency preparedness, and daily health activities
5. How did you manage health issues during COVID-19? Probes: consultations, allied services, investigations, medications, and self-management
6. How were other elements managed? Probes: transport, finance, and getting help from others
7. How did you manage the period of isolation during lockdown?
8. What did you suggest to improve healthcare access in similar situations?

**Table 2.** Coding tree with themes, categories, and codes

*Themes*	*Risk appraisal and feeling vulnerable*		*Safeguarding against COVID-19*			*Managing routine health care and emergency*				*Pursuing mental and psychological well-being*				
Categories	Appraisal of own fragility and COVID-19 literacy	Fear of losing the partner and zest for healthy longevity	Adherence to COVID-19 prevention measures	Social distancing and limiting outsider exposure	Adopting health-promoting behaviors	Continuing routine health care	Fear of visiting hospital versus managing care	Managing emergency	Harnessing on the available resources and support network	Perennial invisible stress	Rejuvenating spousal companionship	Connectedness with family and friends	Spiritual well-being	Rediscovering self
Codes	Family and friends Multiple chronic illnesses Regularly watched and listened to the news Senior citizens Well literate on pandemic	Fear of infection Getting scared Updates on the pandemic Watched television for news	Following safe hygiene practices Sanitize themselves Avoided socializing	Didn’t open house gate for last two months Don’t venture out Locked inside the house Manage with items available nearby Took leave-without-pay	Doing yoga on terrace Increased household chores Practice aerobics at home Practice breathing exercises Practice knee exercises	Continue with previous medication Home-based diagnostic services Risk in going to hospital Self-monitoring of blood pressure Telecommunication difficult for chronic illness	Doctor lives nearby Nearest pharmacy shop owner helps Teleconsultation	Family physician Emergency queries Time of need Virtual platform Online pharmacy	Ambulances Getting telephone calls from the police Restricted movements into and out Vigilant in the early days	Unique calamity Fear of getting infected Being isolated Multiple alignment Getting depressed Huge media coverage	Help in cooking Help my wife Manage with leftover food More worried for her Play games She feels happy when I am around Wife monitored blood pressure	Daughter pays bill online Make videoconference calls Play group games Relative is a doctor Speak over telephone with neighbors Teach grandchildren	Chant mantras God’s grace is there Home remedies Perform ownership Priest blessed	‘Ramayan and Mahabharat’ re-telecasted Gardening Good old days Relive their youth Watching Old era soap operas

### Data management and analysis

All IDIs were digitally recorded, transcribed into the original language ‘Odia’, and translated to English. Each interview ranged between 35 and 45 min. We used the inductive approach of thematic analysis (Miles *et al.*, 2014). The meaning units, parts of the transcripts, which were related to the purpose of the study were identified through an open coding process; two authors (SD, KCS) coded the data and the inter-coder reliability (code frequency correlates to 86%) was checked by PM. After the initial coding for four IDIs, the team discussed and reached at a consensus on a particular coding tree. After completion of coding, the code wise output was extracted and read thoroughly by the research team and then the themes were consultatively derived. MAXQDA software (MAXQDA Analytics Pro 2020, VERBI GmbH Berlin, Germany) was used for coding of the text segments and categorization.

Several steps were followed to ensure the credibility of our results – memos were used during the analysis process; the interim findings were repeatedly discussed and deliberated among the study team for interpretation and conceptualization of themes. During the analysis, the digitally recorded versions and the Odia transcript and the English transcripts were cross-checked repeatedly during the coding process in order to understand the appropriate meaning of the text (Van Nes *et al.*, 2010). We further debriefed the findings with two participants after preliminary analysis and solicited their inputs (member check). The Consolidated Criteria for the Reporting of Qualitative Research (COREQ) guideline was used for reporting of the study (Booth *et al.*, 2014).

### Ethical considerations

The study was approved by the Institutional Ethical Committee of the XXXX (XXXX/XXXX). Those willing to participate in the study were interviewed through a virtual platform; the participation was purely voluntary. Before proceeding with the scheduled interview, verbal consent was obtained and recorded telephonically. All interviews were digitally recorded with prior permission. All the participants were assured that confidentiality and privacy of the information would be maintained at every step and the results would be published anonymously.

### Findings

The findings entail the analysis of interviews of 11 elderly couples. The average age of the interviewees was 67, ranging from 60 to 85 years. Hypertension was the most reported common condition (*n* = 17) followed by diabetes (*n* = 11). Some had other illnesses, namely chronic obstructive pulmonary disease, chronic kidney disease, cardiac disease, eye disorders, osteoarthritis, and neurological problems.

The textual and interpretive analysis yielded four major themes: (1) Risk appraisal and feeling vulnerable; (2) Safeguarding against COVID-19; (3) Managing routine health care and emergency; and (4) Pursuing mental and psychological well-being (Table 2).

### Theme 1: risk appraisal and feeling vulnerable

The couples were well aware of their frailness, heightened susceptibility to the COVID-19, and the consequential severity. The self-risk appraisal and fear of losing own or partner’s life infused a sense of urgency to counteract the situation by adopting multiple strategies.

Category 1: Appraisal of own fragility and COVID-19 literacy

Interviewees had information in prior regarding the impending COVID-19 outbreak and were well literate of the ongoing pandemic. They regularly followed the news in electronic media, discussed over phone with family and friends to be updated. Further, they knew that elderly and having multiple chronic illnesses had a higher risk for COVID-19 infection and complications.

“If I contract with the COVID virus, I will be in a critical situation. I have had poor immune status, I have had bypass surgery, and since 35 years I have had diabetes, which are high risk factors. We’re afraid because we’re senior citizens, and having multiple diseases.” (Husband, Couple 7)

“I can’t manage myself I’m 70 years old. I told my husband that if anything happens, I can’t take you to the doctor alone.” (Wife, Couple 5)

Category 2: Fear of losing the partner and zest for healthy longevity together

They were anxious not only for their health and well-being but for each other too. Since they were residing alone, their greatest fear was if either one of them would get the infection and be hospitalized. The very thought of getting separated from each other and the adverse unforeseen circumstances was a constant source of anxiety. Some of the couples expressed that they too had an equal desire to live as anyone younger. The couples also realized that they would have to manage this challenging situation together on their own.

“We are now 65 years old, but we still wish for a longer life together; hence we are not ready to take any risk.” (Husband, Couple2)

“Though I am worried for myself, I am more concerned about her (i.e. wife’s) health; as she is diabetic, hypertensive and asthmatic.” (Husband, Couple 10)

### Theme 2: safeguarding against COVID-19

The elderly couples took necessary precautions to avoid any possible source of COVID-19 exposure and adopted different health-promoting practices for better physical strength and immunity.

Category 1: Adherence to COVID-19 prevention measures

Many of the couples were in touch with public instructions for the elderly and followed meticulously the prescribed COVID-19 prevention norms and behaviors. Barring essential visits, they primarily stayed inside the house, and avoided socializing and shopping. Whenever they went out, upon returning, they took diligent care to sanitize themselves.

Category 2: Social distancing and limiting outsider exposure

The couples reduced social interaction with neighbors and all friendly home visits. Rather, they preferred to be in telephonically connected with all. Their housing society also imposed restrictions on visitors and household helps. They also withheld all outdoor physical activities like morning exercise and walks with friends. Many of them opined that they were slowly getting out of touch with their outdoor healthy lifestyle practices.

“Our residential complex society has restricted the domestic helps and vendors’ entry. So, no one comes here. We are also not allowing outsiders to enter into our house, not even if they wear a mask.” (Husband, Couple 10)

Category 3: Adopting health-promoting behaviors

Many couples believed that they needed to maintain and promote good physical health. Hence, they found ways to resume their physical activity and diet plans. Instead of going out, they did exercising and walking within the house. Traditional health practices like yoga were also followed by many. Some of the participants mentioned using herbal and home remedies to boost their immunity.

“I walk around the terrace of the house. After walking, I feel satisfied. My blood sugar is in balance as I am walking every day."…I used to attend Yoga classes, but since the centre has been closed now, I am doing it daily at our roof top terrace.” (Wife, Couple 6)

“I drink only warm water throughout the day. I don’t drink chilled water at all. I don’t know how effective it is, but there is a home grown herb Tulsi (The Holy Basil). I add a pinch of its leaves to the warm water and drink it in the early morning for better immunity.” (Wife, Couple 6)

### Theme 3: managing routine health care and emergency

Category1: Continuing routine health care

Many couples reported having multiple chronic illnesses for which they were undergoing frequent consultations, ‘to keep the illness under check’. They also were under supportive procedures like physiotherapy. During COVID-19, most of the couples avoided regular consultations and one participant taking cancer treatment outside the state decided to consult a local specialist and continued treatment. They preferred to take medical consultations only when absolutely necessary. A few also pointed out that their primary physician might not be available, and they will be treated by someone who does not know about their illness.

“If you go to a doctor, then the doctor is not in a position to examine you properly and he may not come out with a proper diagnosis and try to prescribe medicines just out of the experience, without properly examining you as its COVID time.” (Husband, Couple 8)

Category 2: Fear of visiting hospital versus managing care

The couples were also wary of getting infected at the hospital, and they presumed, ‘hospitals are getting COVID-19 patients only’. Some interviewees reported the dilemma of balancing the fear of having an untreated illness and the fear of getting a COVID-19 infection while visiting the hospital. They tried to manage their illness by themselves. Many with stable illnesses continued the same medications.

“I heard that when a person was tested positive and hospitalized by COVID-19, he/she was only treated for COVID-19. They have ignored other chronic illnesses that we have… that might make our condition worse, so we avoid hospital visits, take more precaution to prevent COVID.” (Wife, Couple 9)

Category 3: Managing emergency

Some of the couples reached out to their family physician, who was well aware of their health conditions. The couples expressed gratitude while describing their physicians, who often responded to their emergency queries and guided their treatment at the time of need. Most of the consultations were done by virtual platforms with video calls where prescriptions were shared. Respondents took the help of local or online pharmacies for medicines and neighborhood laboratories for home sample collection with adequate precautions.

“No, I don’t go to anyone else. I only go to him. He is like our family doctor. He knows everything about us. I go to him only.” (Wife, Couple 6)

A participant pointed out that:

“We are being advised for telemedicine, but there is a challenge. I feel you can consult over the phone for a new problem, but it might be difficult to explain about a chronic problem to an unknown physician.” (Husband, Couple 10)

Many of the participants feared an emergency health situation when they have to go to the hospital. Some of them reported being prepared for such an event. They explained that the government is providing enough support like making available ambulances, hired transport, and helplines. ‘All these services prioritize older persons’, one of the couples remarked. In one instance, a wife said about calling up the helpline and received help in transporting her husband to the hospital. Many of those interviewed suggested that home-based care for the elderly can be helpful in such situations.

Category 4: Harnessing available resources and support network

Local communities and residential societies were very helpful to the elderly couples. They also ensured that the residents, particularly the elderly, were supported for their basic needs and medicines. All the interviewees also said about news agencies also sharing information on essential government services like ambulances, teleconsultation platforms, and police.

For instance, few respondents highlighted the support of the local pharmacy in the home delivery of drugs. At the same time, a few acknowledged the support of a physician in the neighborhood.

“ My neighbor who is an employee of the bank helps me with finance. I gave him a cheque, and he brought cash for me. We have been handling this cash until now.” (Husband, Couple 10)

Some of the couples reported getting telephone calls from the police during the pandemic enquiring about their well-being. They were encouraged to ask for help of any kind when needed using the helpline numbers.

“The local police are very helpful. When I rang them for something and asked them to find out about it, they responded immediately.” (Wife, Couple 6)

### Theme 4: pursuing mental and psychological well-being

Category 1: Perennial invisible stress

Some participants expressed that the COVID-19 pandemic is different from other calamities they have faced in their lifetime. They explained that they had to live with the fear of the virus all the time. The fear of getting infected, being isolated when positive, or the fear of death of themselves and their loved ones were stressful. Many with multiple ailments also reported that they apprehended an emergency health condition ‘in the back of their mind’ all the time. Many felt lonely when not able to meet close ones. Some spouses reported their partner to be ‘depressed’ in the initial days.

“We have seen cyclones and floods many times, but this is something different. Those caused lots of destruction with loss of property and lives. Here we have to live with the fear of an unseen virus all the time. We can’t even think of normal life, and we don’t know when this will end. No one will come for our help also if we get the disease.” (Husband, Couple 2)

Although they found the news reports related to COVID-19 informative, but at the same time overwhelming. They selectively listened to news to avoid repetitive negative reinforcement lest it might aggravate their COVID-19 anxiety and apprehension.

“Sometimes we are scared to hear the huge coverage of COVID-19 news, in particular the repeated message ‘older is risky’, although the message is useful.” (Wife, Couple 7)

“Now I’m avoiding television because of the depressing news.” (Husband, Couple 7)

Category 2: Rejuvenating spousal relationship

Besides caring for each other, many spouses reported enjoying their partners’ company. They engaged in conversations, reminisced memories, watched TV together, and shared household tasks. Some of the couples reported cooking together or having a stroll in the garden.

“I am helping my wife in *the kitchen—cutting vegetables and washing utensils* and spending some quality time with her, which otherwise I wasn’t doing.” (Husband, Couple 8)

In some couples, where one of the partners was dependent, their spouses took the utmost precautions not to expose them to outsiders. They managed all chores as well as the responsibility of their partner. They carried out basic examinations like measuring blood pressure, testing blood sugar levels, or giving insulin injections.

“She helps a lot, she cares about everything—food, medication, and other basic needs. The most significant support is to monitor my blood pressure twice daily regularly.” (Husband, Couple 4)

Category 3: Connectedness with family and friends

A majority with children staying away was already familiar with social media platforms. They increased the use of digital technology during the pandemic and were in regular touch with family members, and at times were engaged remotely with their grandchildren.

“My granddaughter asks her mathematics doubts over the phone. And grandson also asks to be taught over the phone, he is in class III, I enjoy coaching them and clarifying their doubts.” (Husband, Couple 9).

“I speak to my sisters over the phone… My mother is alive, so I talk to her; I talk to my brother and sister-in-law, and my sisters. I talk with them over the phone.” (Wife, Couple 6)

“Every 2-3 days there is a video conference with the children and grandchildren altogether, so that continues for some 30–45 min, then making calls to the relatives who are in the city to have chitchat over the phone.” (Husband, Couple 9)

Category 4: Spiritual well-being

Many of the couples resorted to spiritual paths to alleviate their fears and enhance their lives. Faith in God, acceptance of the work of destiny, and belief in fate helped many to be in peace with oneself. They reported following rituals like and ‘chanting mantras’ (Wife, Couple 5) and agreed that ‘having faith in God and believing in destiny gave them the strength to endure’ (Wife, Couple 6). Some of them reflected on their lives and rediscovered a new meaning or purpose in their lives. Many of them learned to value family and spousal relationships complementing each other.

“I sit comfortably and chant Mahamrutyunjaya Mantra. While performing puja I read Bajrangbali’s mantras. While doing all these, I get a hope that God might protect us from this problem.” (Wife, Couple 5)

Category 5: Rediscovering self

Many of the participants rediscovered their past recreational activities. They found immense joy in reliving their memories by engaging in activities like reading books or gardening that they once loved but never found time to pursue. Watching television was touted as refreshing by a majority of the participants. However, watching old era soap operas that were re-telecasted on television was considered to be most engaging as they believed that it helped them relive their youth and took them back to the memories of their good old days.

“Television, since Ramayana and Mahabharata are being re-telecasted, that too four shows a day, so that engages us for four hours every day. In the morning we listen to old songs for 2 h.” (Husband, Couple 9)

“I’m also gardening, reading classic literature and playing music that I haven’t been able to do before the Pandemic; I’m doing it now because of COVID-19. (Husband, Couple 11)

## Discussion

To the best of our knowledge, this is the first study in the region, which explored the various ways through which elderly couples living alone managed and coped with the COVID-19 pandemic. Although initially, the older couples were inundated with fear, anxiety, and apprehensions; slowly with time, most of them had learned to adopt and adapt with the situation. The couples were able to demonstrate a resilient response to the COVID-19 phenomenon by converting adversity to their advantage with a certain extent of learning, unlearning, and relearning (Domajnko & Pahor, 2015).

**Figure 1.**
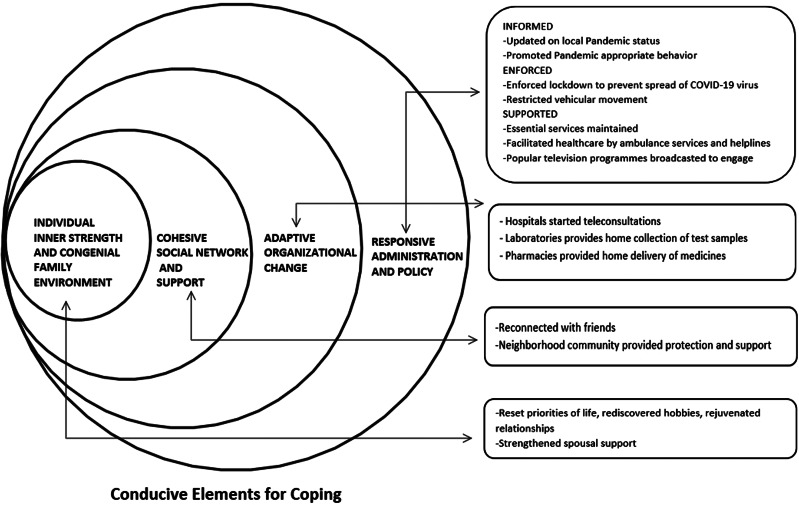
Elements conducive for coping at different levels during the pandemic

Resilience though primarily driven by inner motivation is continually shaped by the cultural context and socio-ecological environment (Panter-Brick & Eggerman, 2012). We too found different contributory factors at the individual, family, community, organizational, and system levels, thus enabling the honing of coping responses. These conducive elements at different levels during the pandemic are summarized in the conceptual framework (Figure 1).

As seen, the assistance of the spouses, especially attitude of complementing each other, strong digital connectivity with their families and relatives, the formal and informal support of different essential services organizations, community networks like local residential society, neighborhood pharmacy, and diagnostic laboratories, relationship with the family physician, and timely response from public helpline system have been critical to meet their routine as well as emergency needs.

The couples were well aware of their vulnerability to the pandemic and began to take countermeasures by engaging in self-protective and health-promoting behavior (Ågård *et al.*, 2015). Apart from strict adherence to all COVID-19 precautionary measures to safeguard, they also followed self-health monitoring, indoor physical activity, practice of yoga and breathing exercise to prescribed medications, balanced diet to stay healthy, and avoid falling sick. They reduced their encounters with outsiders, paid attention to hygiene and sanitation practices, and even changed their health-seeking behavior for routine illnesses; similar to previous findings (Wolf *et al.*, 2020; Neumann-Podczaska *et al.*, 2020).

They also took efforts to be mentally healthy. Given the COVID-19 information overflow dominating all news and social media, they filtered by selective listening. The couples typically avoided all such news or media information, which induced fears about the illness and reinforced apprehension, as they did not want their own COVID-19 anxiety levels to escalate (Jungmann & Witthöf, 2020). Interestingly, as with many elderly couples residing alone, certain factors facilitated the coping responses to the pandemic situation (Wong *et al.*, 2020; Chew *et al.*, 2020; Justo-Alonso *et al.*, 2020; Halperin, 2020). First, most elderly couples in urban areas were retired and financially stable and did not have to worry about earning or livelihood during the lockdown (Son *et al.*, 2020; Heath *et al.*, 2020). Thus, they had no compelling necessity of going out of the house and were able to make informed choices to stay at home. Further, as they were living alone, the risk of getting an infection from a younger and socially active family member was not there. It is mostly seen that close exposure to working-age households or family members puts the homebound elderly on a higher risk of COVID-19 infection (Prem *et al.*, 2020). Though ‘*staying alone’* has its own associated challenges, but at the same time under the prevailing situation, it resulted in ‘*staying protected’* for the older couples as well (Padyab *et al.*, 2019).

At the family level, they received psychological and physical support from their spouses and enjoyed work-sharing in order to cope with the increased stress of household chores in the absence of housework assistance (Haslam, 2020). Chronic disease management requires a significant effort to change lifestyles, and a spouse plays a key role in managing the health needs of patients with chronic diseases (Ågård *et al.*, 2015; Pati *et al.*, 2021). Furthermore, being adept at using technology, they could rely on digital connectivity to reach out to their friends and family and were able to compensate for in-person social contact by virtual interactions with friends and family (Nicol *et al.*, 2020).

At the community and organizational level, our participants were supported by local pharmacy and diagnostic services, and teleconsultation. Community pharmacists are the most accessible health workers to the common people and have a lot to offer in the context of the COVID-19 response. This has led to significant changes in the health systems of many countries (Hedima *et al.*, 2020; Goodman-Casanova *et al.*, 2020). Ensuring the continuation of treatment through door-step delivery of drugs and consumables to the elderly is crucial. Community pharmacists should learn the skills of elderly preventive care and to develop emergency pharmaceutical care plans for this population (Tipnis & Hiremath, 2008). Our findings suggest that future infectious disease outbreaks or the public health emergency preparedness plans should include in community pharmacy model toward the care continuum.

In addition, our study found that the community was taking active measures to protect elderly people from exposure. Home delivery of essentials by local grocery shops and neighbors, home delivery of medications from neighborhood pharmacies, door-step diagnostic services, and door-step banking facilities are few instances where the community responded to the primary needs of the elderly. Older people who have a strong community and system support exhibit a better response to adverse circumstances. Similarly, a study in the United States of America observed that the elderly who had a well-built social support system demonstrated stronger resilience than those who managed their own without much perceived support (Killgore *et al.*, 2020; Armitage & Nellums, 2020).

Considering the older people’s vulnerability to the pandemic, the government gave health advisories in media, which enabled them to learn about their heightened risk (Hebbar *et al.*, 2020; Kumar *et al.*, 2020). The fear of contagion prompted them to adhere to the government’s directives. The health directives could also successfully induce sensitivity toward the elderly needs in the community. Furthermore, allowing the pharmacy and other essential item shops to operate gave them a window to provide home-based services to the older population. Besides, the decision to re-telecast the popular old-time soap operas was instrumental in enabling the elderly to relive their youth days and maintain their psychological well-being during the pandemic.

While aging is an inevitable and inescapable phase of transition, physical activity has proven to be a useful tool for addressing age-related problems. Consequently, emerging patterns of physical activity and inactivity demand close attention (Gour *et al.*, 2020). In our study, indoor exercise such as ‘yoga and pranayama’, light exercise and engagement in household chores, gardening were perceived as alternating strategies to replace outdoor exercise during the COVID-19 pandemic. As in previous literature, yoga and meditation can be conceptualized as a family of complex emotional and careful regulatory practices for psychological and physical health (Yang *et al.*, 2020).

There is growing evidence to indicate that a possible health risk level for low exercise or inactivity. A previous longitudinal qualitative research in India explored the effect of yoga/light exercise; where participants experienced improved strength and flexibility, enhanced sleep quality, energy level, and social behavior (Gour *et al.*, 2020). Therefore, the future geriatric health care plan should emphasize the indoor activities, including ‘yoga and pranayama’ as well as light exercise.

Although older people are thought to be fragile, they have stronger psychological resilience as witnessed in our study (Gooding *et al.*, 2012). Many participants were comfortable and secure being at home and expressed willingness to live under lockdown restrictions for a longer period of time to avoid risking their lives. This determination could be considered as a positive sign that could probably make them sail through the emotional turmoil they would otherwise encounter if the pandemic continued for a longer period of time (Djalante *et al.*, 2020; Kavčič *et al.*, 2020). On the other hand, they creatively used the time by pursuing their past leisure time activities. This path to rediscovery helped them to lead a new normal life and infused positive thinking.

### Methodological considerations

In an effort to enhance the trustworthiness of the study, we adopted research and source triangulation. To ensure the conformability of the study findings, three authors inter-checked the coding, themes, and descriptions. The authors have diverse educational and professional backgrounds, such as psychiatry, public health, medical anthropology, behavioral sciences, health policy, and planning with previous experience of aging research, which helped in broadening the analytical dimensions and interpretation of findings. The balanced male–female composition of the team took care of the gender perspective of the analysis. Transferability to other settings was appraised through providing the detailed study design, sampling strategy, data collection, and analysis method. The results can theoretically be generalised only for urban middle-income elderly couples living without any other family members in an LMIC context during a similar public health lockdown situation.

## Conclusions

Our findings indicate spousal support and social network with adaptive organizational change and a responsive public system to be crucial in mitigating the older adults’ challenges in a pandemic. Elderly patients’ activation to meet their chronic care needs with less dependence on the health system, and engagement with health promotion may be encouraged. Digital technology-enabled health care appears promising for older population too. Health system preparedness plan should consider including geriatric health and preferably community pharmacy model, family-centric, and home-based chronic care delivery models during a public health emergency. Future studies on coping response of elderly couples living alone in urban slums and rural settings may provide further insights.
